# Supplementary figures and images for: Soil respiration of a Moso bamboo forest significantly affected by gross ecosystem productivity and leaf area index in an extreme drought event
Source: PeerJ. 2018 Oct 31;6:e5747. doi: 10.7717/peerj.5747 (PMC6215440; doi:10.7717/peerj.5747)

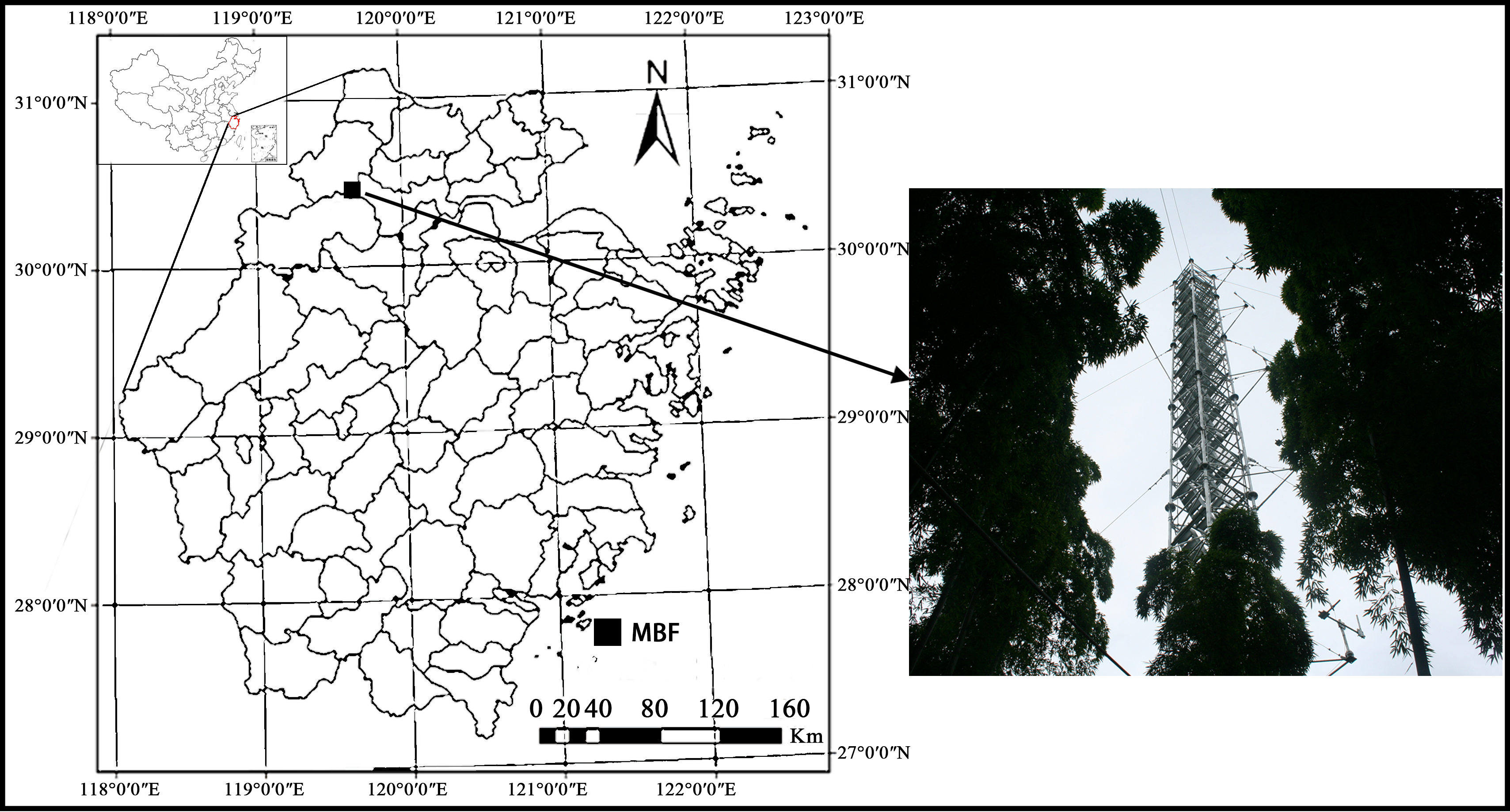

Supplement: Supplemental Information 1 — Black square in the Moso bamboo forest (MBF) flux tower site. Photo by Huaqiang Du. [file peerj-06-5747-s002.png]
